# Low population serum microneutralization antibody titer against the predominating influenza A(H3N2) N121K virus during the severe influenza summer peak of Hong Kong in 2017

**DOI:** 10.1038/s41426-018-0041-1

**Published:** 2018-03-06

**Authors:** Houshun Zhu, Andrew C. Y. Lee, Can Li, Winger W. N. Mak, Yetta Y. Chen, Kwok-Hung Chan, Anna J. X. Zhang, Wai-Fong Fung, Rui-Qi Zhang, Yim-Fong Fung, Rosana W. S. Poon, Joy-Yan Lam, Sidney Tam, Ivan F. N. Hung, Honglin Chen, Kwok-Yung Yuen, Kelvin K. W. To

**Affiliations:** 1 Department of Microbiology, Li Ka Shing Faculty of Medicine, The University of Hong Kong, Hong Kong Special Administrative Region, China; 2 Department of Microbiology, Queen Mary Hospital, Hong Kong Special Administrative Region, China; 3 State Key Laboratory for Emerging Infectious Diseases, Li Ka Shing Faculty of Medicine, The University of Hong Kong, Hong Kong Special Administrative Region, China; 4 Research Centre of Infection and Immunology, Li Ka Shing Faculty of Medicine, The University of Hong Kong, Hong Kong Special Administrative Region, China; 5 Division of Clinical Biochemistry, Department of Pathology, Queen Mary Hospital, Hong Kong Special Administrative Region, China; 6 Department of Medicine, Li Ka Shing Faculty of Medicine, The University of Hong Kong, Hong Kong Special Administrative Region, China

## Abstract

The 2017 Hong Kong influenza A(H3N2) summer season was unexpectedly severe. However, antigenic characterization of the 2017 circulating A(H3N2) viruses using ferret antisera did not show significant antigenic drift. We analyzed the hemagglutinin amino acid sequences of A(H3N2) virus circulating in Hong Kong in 2017, and found that viruses with hemagglutinin N121K substitution, which was rare before 2017, emerged rapidly and dominated in 2017 (52.4% of A[H3N2] virus in 2017 contains N121K substitution). Microneutralization assay using archived human sera collected from mid-2017 showed that the geometric mean microneutralization titer was 3.6-fold lower against a 2017 cell culture-grown circulating A(H3N2)-N121K virus (3391/2017 virus) than that against the cell culture-grown 2016–2017 A(H3N2) seasonal influenza vaccine-like vaccine virus (4801/2014 virus) (13.4 vs 41.8, *P* < 0.0001). Significantly fewer serum specimens had a microneutralization titer of 40 or above against 3391/2017 virus than that against 4801/2014 virus (26.4% vs 60.0%, *P* < 0.0001). Conversely, the geometric mean hemagglutination inhibition titer was slightly higher against 3391/2017 virus than that against the 4801/2014 virus (96.9 vs 55.4, *P* < 0.0001). Moreover, 59.1% of specimens had a significantly lower microneutralization antibody titer (≥4-fold) against 3391/2017 virus than that against 4801/2014 virus, but none for hemagglutination titer (*P* < 0.0001). Similar results of microneutralization and hemagglutination titers were observed for day 21-post-vaccination sera. Hence, the 2017 A(H3N2) summer peak in Hong Kong was associated with a low-microneutralization titer against the circulating virus. Our results support the use of microneutralization assay with human serum in assessing population susceptibility and antigenic changes of A(H3N2) virus. Novel and available immunization approach, such as topical imiquimod followed by intradermal vaccination, to broaden the neutralizing antibody response of influenza vaccine should be considered.

## Introduction

Seasonal influenza virus poses a significant burden to human health^1^. Humans are susceptible to seasonal influenza virus infections because of the continuous antigenic changes of influenza virus. These antigenic variants are caused by mutations in the viral surface glycoproteins hemagglutinin and neuraminidase, which allow the virus to escape from pre-existing humoral immunity induced by prior natural infection or vaccination^2^.

Studies involving human volunteers experimentally infected with live influenza virus have shown that hemagglutination inhibition titer of 40 or above correlated with a protection of about 70%^3, 4^. A recent study has shown that microneutralization titer induced by vaccination correlates with protection, in which a microneutralization titer of 40 or above was associated with 60% protection^5^. Hence, the World Health Organization (WHO) determines antigenic drift of circulating influenza viruses by analyzing the hemagglutination inhibition titer using post-infection sera collected from ferrets infected with the vaccine virus strain, or with post-immunization sera from human vaccinees^6, 7^. Virus neutralization assay is also performed on some strains to supplement the result from hemagglutination inhibition assay, especially for the recent A(H3N2) strains that are found to have poor agglutination with red blood cells^7^.

In 2017, an unexpectedly high-summer peak of influenza A(H3N2) occurred in Hong Kong. The number of hospitalizations and deaths due to A(H3N2) in 2017 summer influenza season was similar to those in 2014–2015 winter season^8^. A similar severe summer influenza epidemic also occurred in Taiwan^9^. Unlike the 2014–2015 winter epidemic, which was associated with an antigenically drifted A(H3N2) virus^10^, there was no significant antigenic changes for the circulating A(H3N2) viruses in 2017. The WHO and the Center for Disease Control reported that circulating A(H3N2) viruses in 2017 were well-inhibited by ferret antisera raised against cell culture-propagated vaccine viruses (A/Hong Kong/4801/2014 [4801/2014] or similar viruses) recommended for the 2016–2017 northern hemisphere influenza season^7, 11^.

One possible explanation for the unusually severe A(H3N2) epidemic in 2017 was poor vaccine effectiveness conferred by the recommended influenza vaccine for the 2016/2017 northern hemisphere influenza season^7, 11–14^. The poor vaccine effectiveness for A(H3N2) has been attributed to mutations arising from egg-grown vaccine viruses^15, 16^. Ferret antisera raised against egg-grown vaccine viruses have low-microneutralization titers against the circulating cell culture-grown A(H3N2) viruses^16^. Human serum panels from vaccinees showed a lower geometric mean hemagglutination inhibition and microneutralization titer against cell culture-grown circulating A(H3N2) viruses than those against the egg-grown vaccine virus^7^. Based on these data, the WHO has changed the recommendation for the vaccine to be used in 2018 southern hemisphere to A/Singapore/INFIMH-16-0019/2016 (H3N2)-like virus, in which even the egg-propagated virus can elicit good antibody response against the circulating strains.

In addition to poor vaccine effectiveness, other non-vaccine related factors may also affect the size of an epidemic. This study sought to assess the humoral immunity of the general population against the circulating A(H3N2) virus in 2017. We specifically used cell culture-grown viruses to avoid unwanted mutations that can be generated during virus passage in eggs. We have found that the geometric mean microneutralization titer was significantly lower against a representative 2017 circulating A(H3N2) virus than that against a pre-2017 A(H3N2) virus, suggesting that there is reduced protection conferred by the humoral immunity generated by prior natural wild-type virus infection or by immunization with egg-based influenza vaccine.

## Results

**Fig. 1:**
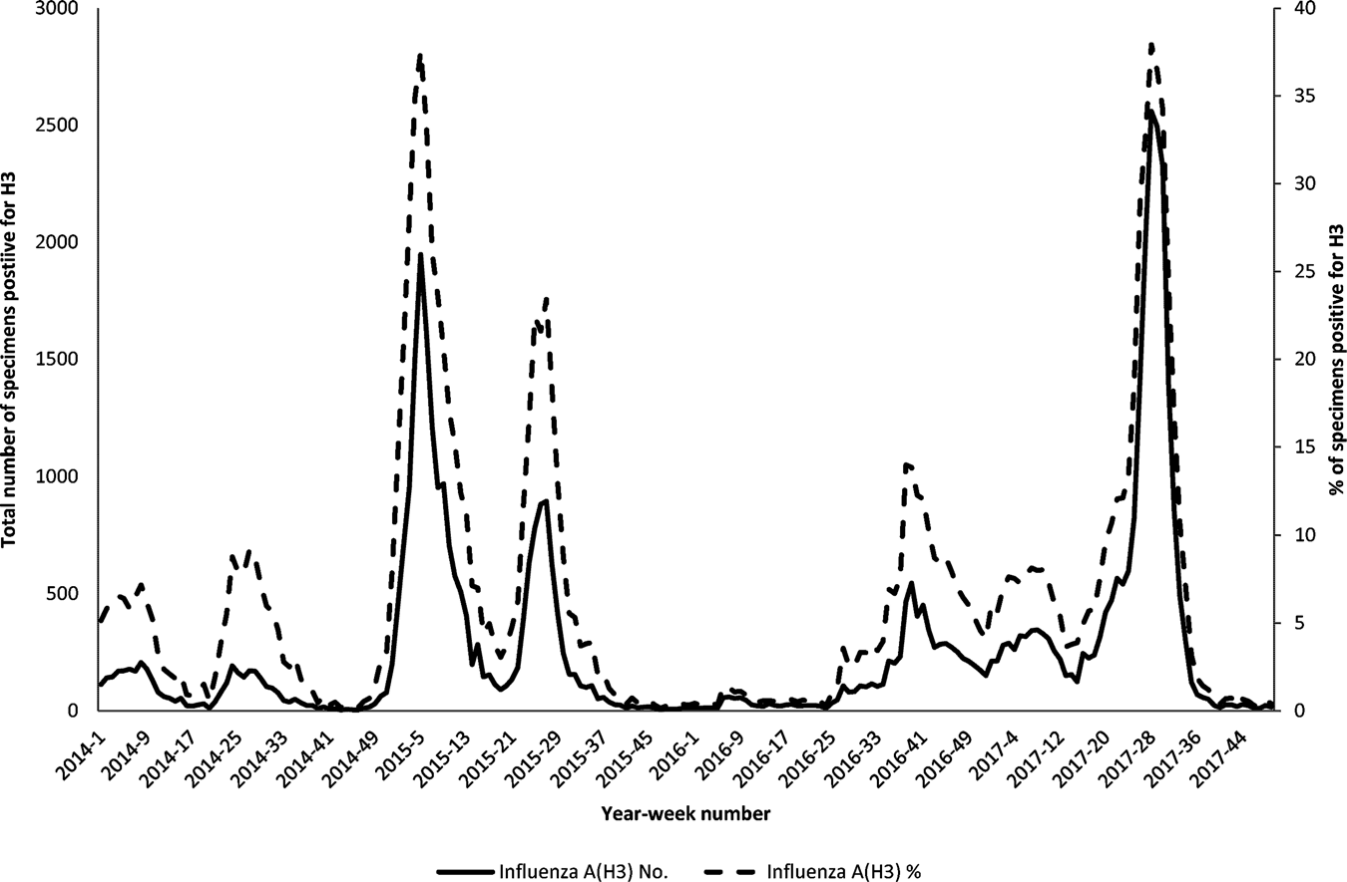
Total number and percentage of influenza A(H3) virus detected in Hong Kong from 2014 to 2017. The data were obtained from the Public Health Laboratory Services of Hong Kong^39^

The surveillance data of influenza A(H3N2) virus in Hong Kong from the Public Health Laboratory Services Branch showed that the number of influenza A(H3)-positive clinical specimens in 2017 summer influenza season was similar to that of 2014–2015 winter influenza season (Fig. 1).

**Fig. 2:**
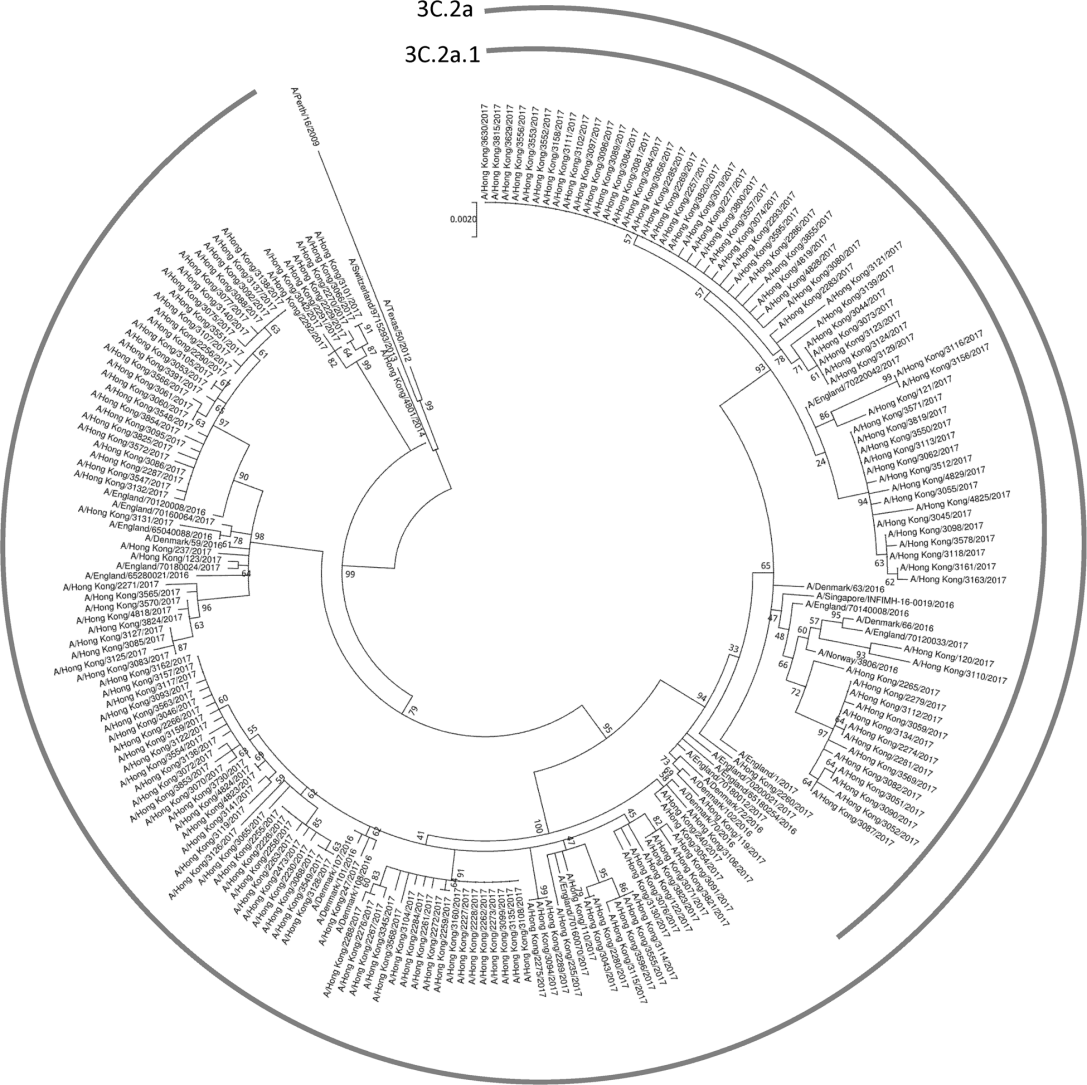
Phylogenetic trees showing the relationship between influenza A(H3N2) viruses from Hong Kong in 2017, other representative A(H3N2) viruses, and recommended A(H3N2) viruses for influenza vaccines^12, 13^. The phylogenetic tree was constructed using the Maximum Likelihood method based on the Hasegawa–Kishino–Yano model. All sequences were obtained from the Global Initiative on Sharing All Influenza Data (GISAID) Database (Supplementary Table S1)

Next, we analyzed the hemagglutinin amino acid sequence data from viruses collected in Hong Kong, which have been deposited into the GISAID EpiFlu database as of 13 December 2017 (Supplementary Table S1). Comparison of viruses collected in 2017 and in 2014–2016 showed that three substitutions at antigenic site A of the hemagglutinin (N121K, T135K, and S144K) have rapidly emerged in Hong Kong (Table 1). The proportion of A(H3N2) viruses with N121K substitution increased from 0% (0/51) in 2014/2015 and 7.7% (1/13) in 2016, to 52.4% (99/189) in 2017; T135K substitution increased from 0% (0/64) from 2014–2016 to 29.1% (55/189) in 2017; and S144K substitution increased from 0% (0/64) from 2014–2016 to 19.6% (37/189) in 2017. For antigenic site B, a novel F193S substitution was found, but was only present in 1.6% (3/189) of the 189 Hong Kong isolates characterized in 2017. Phylogenetic analysis of the hemagglutinin gene showed that all A(H3N2) viruses in 2017 from Hong Kong belongs to genetic clade 3C.2a, with 39.7% (75/189) within subclade 3C.2a1 (Fig. 2).

**Table 1:** Major amino acid substitutions of A(H3N2) viruses from Hong Kong. Amino acid sequence data were downloaded from Global Initiative on Sharing All Influenza Data (GISAID) EpiFlu database (Supplementary Table 1)

HA amino acid substitution	2014–2015 (*n* = 51)	2016 (*n* = 13)	2017^a^ (*n* = 189)
N121K	0 (0%)	1 (7.7%)	99 (52.4%)
T135K	0 (0%)	0 (0%)	55 (29.1%)
S144K	0 (0%)	0 (0%)	37 (19.6%)

^a^ Up to 13 December 2017

A(H3N2) viruses with N121K substitution were also found to be present in many A(H3N2) viruses from other countries^12, 13^. A previous study from Denmark has shown that cluster 3C.2a/N121K or 3C.2a1/N121K had lower vaccine effectiveness than clusters without N121K^12^. Therefore, we have chosen a 2017 A(H3N2) virus, 3391/2017 virus, which possesses N121K substitution for the subsequent microneutralization and hemagglutination inhibition assays.

Randomly selected archived serum specimens taken from 435 hospital in- and out-patients not symptomatic for influenza or any acute respiratory illness were tested by microneutralization and hemagglutination inhibition assay against the 2017 circulating A(H3N2) virus (3391/2017 virus), and a pre-2017 A(H3N2) virus from Hong Kong (4801/2014 virus), which is antigenically similar to A(H3N2) viruses in the 2015–2016 northern hemisphere influenza seasons^17^. The geometric mean microneutralization titer was 3.6-fold lower against 3391/2017 virus than that against 4801/2014 virus (13.4 vs 47.8, *P* < 0.0001) (Table 2). The proportion of individuals with a microneutralization titer ≥40 was also significantly lower for 3391/2017 virus than 4801/2014 virus (26.4 vs 60.0%, *P* < 0.0001). However, the geometric mean hemagglutination inhibition titer was 1.7-fold higher against 3391/2017 virus than 4801/2014 virus (96.9 vs 55.4, *P* < 0.0001), and the proportion of individuals with hemagglutination inhibition titer ≥40 was significantly higher for 3391/2017 virus than that of 4801/2014 (88.3% vs 73.1%, *P* < 0.0001). The percentage of individuals with at least fourfold lower 3391/2017 virus MN titer than 4801/2014 virus MN titer was 59.1% (257/435), while none of the individuals have fourfold lower 3391/2017 than 4801/2014 virus HI titer (*P* < 0.0001) (Table 3).

**Table 2:** Microneutralization and hemagglutination inhibition titer against the 2017 H3N2 circulating strain (3391/2017) and the 2016/17 influenza vaccine A(H3N2) strain (4801/2014)

	Hospital patient (*n* = 435)		*P-*value	Vaccinees (*n* = 200)		*P-*value
Median age (IQR)	66 (43–78)			56 (45–64)		
Virus strain	3391/2017^a^	4801/2014^b^		3391/2017^a^	4801/2014^b^	
*GMT*						
MN (95% CI)	13.4 (12.1–14.9)	47.8 (40.6–56.3)	<0.0001	23.1 (18.8–28.2)	125.1 (99.0–158.1)	<0.0001
HI (95% CI)	96.9 (87.0–107.8)	55.4 (50.1–61.2)	<0.0001	276.7 (232.7–329.0)	166.2 (140.7–196.3)	<0.0001
*% of individuals with antibody titer ≥40*						
MN titer ≥40, No. (%)	115 (26.4)	261 (60.0)	<0.0001	86 (43.0)	164 (82.0)	<0.0001
HI titer ≥40, No. (%)	384 (88.3)	318 (73.1)	<0.0001	191 (95.5)	186 (93.0)	NS

*CI* confidence interval, *GMT* geometric mean titer, *HI* hemagglutination inhibition, *IQR* interquartile range, *MN* microneutralization, *NS* not significant

^a^ 3391/2017 = A/Hong Kong/3391/2017 A(H3N2) (2017 circulating strain; 121K/144K)

^b^ 4801/2014 = A/Hong Kong/4801/2014 A(H3N2) (2016/2017 and 2017/2018 vaccine strain; 121N, 144S)

**Table 3:** Comparison of microneutralization and hemagglutination inhibition titers between 3391/2017 and 4801/2014

Antibody titer against 3391/2017^a^ at least fourfold lower than that against 4801/2014^b^	Hospital patients	Vaccinees
MN titer, No. (%)	257 (59.1%)	142 (71%)
HI titer, No. (%)	0 (0%)	3 (1.5%)

*HI* hemagglutination inhibition, *MN* microneutralization

^a^ 3391/2017 = A/Hong Kong/3391/2017 A(H3N2) (2017 circulating strain; 121K/144K)

^b^ 4801/2014 = A/Hong Kong/4801/2014 A(H3N2) (2016/2017 and 2017/2018 vaccine strain; 121N, 144S)

Since previous studies have shown that humoral immunity induced by influenza vaccine derived from egg-grown viruses is poorer against the original virus, we also assessed the antibody titers for day 21-post-vaccination serum samples of recipients of the 2016–2017 seasonal influenza vaccine recommended for the northern hemisphere. Similar to hospital patients, the geometric mean microneutralization titer for post-vaccination sera was 5.4-fold lower against 3391/2017 virus than that against 4801/2014 virus (23.1 vs 125.1, *P* < 0.0001), while the geometric mean hemagglutination inhibition titer was significantly higher for 3391/2017 virus than that against 4801/2014 virus (276.7 vs 166.2, *P* < 0.0001) (Table 2). However, there was no significant difference in the proportion of hemagglutination inhibition titer ≥40 against 3391/2017 or 4801/2014 virus for the post-vaccination sera.

We also compared the antibody titer between the younger (18–64 years) and older (65 years or above) age groups. For the hospital patient cohort, the geometric mean microneutralization titers for younger individuals were significantly lower than those of the older individuals against both 3391/2017 (8.9 vs 18.2, *P* < 0.0001) and 4801/2014 (21.2 vs 87.9, *P* < 0.0001) viruses (Table 4). However, for the vaccinees, the microneutralization and hemagglutination inhibition titers for younger individuals were higher than those of the older individuals, although only the geometric mean microneutralization titer against the 3391/2017 virus reached statistical significance.

**Table 4:** Microneutralization and hemagglutination inhibition titers stratified by age group

Age group	Hospital patient					
	MN titer		*P-*value^b^	HI titer		*P*-value^b^
	3391/2017	4801/2014		3391/2017	4801/2014	
18–64 years (*n* = 186)	8.9 (7.8–10.1)	21.2 (15.43–23.13)	<0.0001	74.5 (65.0–85.5)	48.4 (42.2–55.5)	<0.0001
65 years or above (*n* = 249)	18.2 (15.7–21.2)	87.9 (61.43–92.17)	<0.0001	117.8 (101.1–137.3)	61.2 (53.2–70.5)	<0.0001
*P-*value^a^	<0.0001	<0.0001		<0.0001	0.07	

	**Vaccinees**					
	**MN titer**		***P-*** **value** ^**b**^	**HI titer**		***P***-value^**b**^
	**3391/2017**	**4801/2014**		**3391/2017**	**4801/2014**	
18–64 years (*n* = 154)	27.3 (21.4–34.8)	136.7 (104.3–179.1)	<0.0001	287.2 (234.1–352.5)	175.1 (144.2–211.5)	<0.0001
65 years or above (*n* = 46)	13.1 (9.8–17.5)	93.0 (58.0–149.3)	<0.0001	244.0 (176.6–337.1)	139.7 (100.5–194.2)	<0.0001
*P*-value^a^	0.012	0.051		0.088	0.113	

^a^ 18–64 age group vs 65 or above age group

^b^ 3391/2017 vs 4801/2014

## Discussion

Hong Kong is located in the subtropical area with two influenza peaks per year. The winter influenza peak is usually more severe than the summer peak. However, the 2017 summer influenza season was at least as severe as the 2014–2015 winter influenza season in Hong Kong, despite the absence of a significant antigenic drift that can be detected by ferret antisera raised against the cell-grown A(H3N2) virus recommended for the 2016/2017 northern hemisphere vaccine^6, 11^. This study explored the possible reason for the severe 2017 summer influenza season. Analysis of the hemagglutinin amino acid sequence of all influenza A(H3N2) virus sequences deposited into GISAID EpiFlu database showed that A(H3N2) viruses with mutations at antigenic site A, especially N121K, have rapidly emerged in 2017 in Hong Kong. Using archived human serum samples from hospital patients, the geometric mean microneutralization titer was significantly lower against a 2017 A(H3N2) virus with N121K substitution (3391/2017 virus) than that against the WHO recommended A(H3N2) virus for the 2016/2017 northern hemisphere vaccine (4801/2014 virus). The percentage of serum samples with microneutralization titer ≥40 was significantly less for 3391/2017 virus than that of 4801/2014 virus. Furthermore, the percentage of serum samples with at least fourfold lower titer against 3391/2017 virus than that against 4801/2014 virus was also significantly higher for MN titer than HI titer. Similar results were seen in the serum specimens from post-vaccination cohort. Our results support the hypothesis that antigenic changes of the 2017 circulating A(H3N2) virus was sufficient to escape the pre-existing immune response in the general population against the previous H3N2 virus, which was judged by standard ferret serum to be antigenically similar to presently circulating H3N2 virus.

Substitutions at antigenic site A were previously found to be important for antigenic drift^18, 19^. In particular, amino acid position 145 was found to be one of the 7 important sites associated with major antigenic change. When we compared the A(H3N2) viruses from Hong Kong, we have identified three substitutions (N121K, T135K, and S144K) at hemagglutinin antigenic site A which have rapidly emerged in 2017^20^. N121K substitution was most frequently found, detected in over 50% of viruses collected in 2017. N121K is a major substitution emerging in 2017 A(H3N2) viruses worldwide^6, 12, 13^, and this substitution was found in both the clade 3C.2a and subclade 3C.2a1^9^. T135K can result in loss-of-N-glycosylation site at position 133–135^21, 22^. S144K is very close to the receptor binding site. However, T135K was not present in 3391/2017 virus which was used in our serological assays. Further studies are needed to determine whether these substitutions have led to the changes in microneutralization antibody titers.

Unlike geometric mean microneutralization titer, geometric mean hemagglutination inhibition titer was significantly higher for the 3391/2017 virus than that of 4801/2014 virus, although the difference was <2-fold. Discrepancy between human serum hemagglutination inhibition and microneutralization titer has been well reported^23, 24^. Previous studies have shown that the correlation between hemagglutination inhibition and microneutralization titer was poorer for A(H3N2) than that for A(H1N1) virus^25, 26^. However, the reason for such discrepancy in hemagglutination inhibition and microneutralization titer is not clear. There are several possible reasons. First, a poor microneutralization titer may be related to the effect of internal genes on host humoral immune response. In our previous mice model with A(H7N9) virus infection, microneutralization antibodies were not detected despite the presence of hemagglutination inhibition antibody titer^27^. Experiments with reassortant viruses showed that the internal genes of the A(H7N9) virus was responsible for the lower microneutralization titer. Second, a high hemagglutination inhibition titer may be related to cross-reactive antibodies generated by prior infection by A(H1N1). It has been shown that natural A(H1N1) virus infection can induce cross-reactive hemagglutination inhibition antibodies against A(H3N2) virus in humans^28^. Nachbagauer R et al.^29^ has shown that patients infected with A(H1N1) generates antihemagglutinin antibodies against other influenza A virus subtypes. It is possible that prior A(H1N1) infection, especially during the 2016 A(H1N1) epidemic in Hong Kong, could have induced cross-reactive hemagglutinating inhibition antibodies against the current A(H3N2) virus.

It is interesting to note that the younger individuals (aged 18–64 years) have lower microneutralization titer against both 3391/2017 virus and 4801/2014 virus than the older individuals (aged 65 years or above) in the hospital patient cohort, but not among the vaccinees. One possibility is that more elderly were naturally infected than the younger population^8^. Another possibility is that more elderly have received the influenza vaccination as recommended by the Centre for Health Protection in Hong Kong^30^.

The vaccine effectiveness for the 2016–2017 northern hemisphere influenza season was found to be low for A(H3N2), ranging from −52.1% in South Korea to 43% in the USA^14, 31^. Several studies have postulated that the low-vaccine effectiveness against the presently circulating A(H3N2) may be related to the substitutions found in egg-propagated viruses. During egg passage, influenza virus acquires amino acid substitutions in the hemagglutinin protein, which improve the attachment of the virus to avian-type host sialic acid receptors^32^. Studies in the 20th century have already shown that viruses grown in eggs can be less immunogenic than those that are grown in MDCK cells^33^. Poor efficacy of the 2012–2013 egg-propagated influenza vaccine against A(H3N2) was attributed to three amino acid mutations in the hemagglutinin (H156Q, G186V, and S219Y)^34^. Egg-propagated 4801/2014 virus has a hemagglutinin L194P substitution, which affects the structure of the receptor binding site^15^. Egg-propagated 3C.2a vaccine virus (A/Colorado/15/2014) has a hemagglutinin T160K substitution which leads to the loss of a glycosylation site^16^. In our study, we used mammalian MDCK cell-propagated viruses. The 3391/2017 virus used in this study has T at position 160 and L at position 194 of the hemagglutinin protein. Therefore, the significantly lower microneutralization titer induced against the circulating virus when compared with the vaccine virus cannot be explained by virus mutations previously shown to arise through passaging in chick embryo.

Although microneutralization titer may be a better assay for predicting population susceptibility to A(H3N2) infection, microneutralization assay is technically more demanding than hemagglutination inhibition assay. First, microneutralization assay requires live virus with an accurate virus titer, while hemagglutination inhibition assay can be performed using standard control virus antigens that are provided by the WHO kit. Second, since live virus is used, microneutralization assay must be performed inside biosafety cabinet. Third, microneutralization assay takes at least 2 days, while hemagglutination assay can be completed in a single day.

Though a large number of serum samples have been tested, our study was limited by the collection of samples from one teaching hospital in Hong Kong. Due to the difficulty of getting sufficient volume of archived serum samples from children, no data is available for this age sector.

Our result suggests that a reduction in microneutralization titer against A(H3N2) among the adult population may explain the severe summer influenza season in 2017, despite that there is no major antigenic changes detectable by WHO standard ferret serum. Microneutralization titer may be a better measure than hemagglutination inhibition titer for predicting the population susceptibility, severity of A(H3N2) influenza epidemic, and perhaps the effectiveness of the chosen vaccine. Until more data are available, we recommend that both microneutralization and hemagglutination inhibition assay should be performed. Broadening the antibody response of intradermal influenza vaccine by prior topical imiquimod, a Toll-like receptor 7 agonist, may enhance the vaccine effectiveness against these A(H3N2) viruses with antigenic changes^35^.

## Materials and methods

### Cells

All laboratory procedures were performed according to what we previously reported with minor modifications^35^. Madin-Darby canine kidney (MDCK) cells were cultured at 37 °C with 5% CO_2_ in minimum essential medium (MEM) (Gibco, N.Y., USA), supplemented with 10% fetal bovine serum and 1% penicillin/streptomycin. When MDCK monolayer culture reached about 85% confluency, the cells were trypsinized and seeded in 96-well plates at a density of 2 × 10^4^ cells/well and cultured overnight for the microneutralization assay.

### Influenza viruses

The A(H3N2) virus A/Hong Kong/3391/2017 (3391/2017 virus) was collected from a patient on 10 July 2017. The A(H3N2) virus 4801/2014 was the recommended strain for the 2016–2017 and 2017–2018 northern hemisphere influenza season. Both 3391/2017 and 4801/2014 viruses were kindly provided by the Public Health Laboratory Service of Hong Kong. Influenza viruses were propagated in MDCK cells without any passaging in chick embryo. The hemagglutinin genes of each virus were sequenced to confirm that no mutations were introduced into the hemagglutinin sequence during virus propagation. The hemagglutination titer was determined using 0.5% fresh group O human red blood cells. The 50% tissue culture infective dose (TCID_50_) was determined in MDCK cells in the presence of 2 μg/ml l-1-tosylamide-2-phenylethyl-chloromethyl ketone-treated trypsin (TPCK-trypsin). Aliquots of virus stock were kept at −80 °C until use.

### Human serum

Archived human serum samples collected from July to September 2017, which coincided with the falling phase of the summer peak of A(H3N2) epidemic, were randomly selected from the Clinical Biochemistry Division, Queen Mary hospital. In addition, sera from vaccinees of 2016/2017 northern hemisphere recommended quadrivalent influenza vaccine taken on day 21-post-vaccination from November 2016 to June 2017 were also used in this study. The collected sera were aliquoted and stored at −20 °C until testing to avoid repeated cycles of freeze and thaw. This study was approved by the Institutional Review Board of the University of Hong Kong/Hospital Authority Hong Kong West Cluster (HKU/HA HKW IRB).

### Hemagglutination inhibition assay

Hemagglutination inhibition assay was performed as we described previously with modifications^35, 36^. Serum samples were treated with receptor destroying enzyme (RDE) (Denka Seiken, Tokyo, Japan) overnight at 37 °C to remove non-specific inhibitors. Treated serum samples were heat inactivated at 56 °C for 30 min, and were twofold serially diluted in phosphate buffered saline (PBS) starting from 1:10 dilution. Diluted serum samples were mixed with 4 hemagglutinin units of A(H3N2) virus and incubated at room temperature for 1 h. After incubation, 0.5% human red blood cell suspension was added to the serum-virus mixture and incubated at room temperature for 1 h when the red blood cells were completely set in PBS control wells. The hemagglutination inhibition titer was expressed as the reciprocal of the highest serum dilution that completely inhibited haemagglutination. The hemagglutination inhibition assay was performed in duplicate for each sample, and the hemagglutination inhibition titer was taken as the average of the two titers.

### Microneutralization assay

Microneutralization assay was performed as we described previously^35–37^. Briefly, serum samples were heat inactivated at 56 °C for 30 min, and then serially diluted in twofolds with PBS starting at 1:10 dilution. Serial dilutions of heat-inactivated serum samples were mixed with 100 TCID_50_ of A(H3N2) virus and incubated at 37 °C for 1 h. The serum-virus mixture was then added to MDCK cells and incubated for 1 h. After incubation, the serum-virus mixture was removed, and serum-free MEM containing 2 µg/ml TPCK-trypsin was added to the cells, and was further incubated at 37 °C and 5% CO_2_. Cytopathic effect was determined by examination under inversion microscopy after 72 h of incubation. Culture supernatant was mixed with equal volume of 0.5% human red blood cell to confirm the absence of hemagglutination by virus. The microneutralization titer was defined as the highest dilution of serum that completely inhibited cytopathic effect and absence of hemagglutination in 50% of the wells as described previously^38^.

### Influenza epidemiology and virus sequence data

Laboratory surveillance data of influenza virus from 2014 to 2017 (up to week 49) from the Public Health Laboratory Services Branch was obtained from the Centre for Health Protection website^39^. Gene sequence of hemagglutinin gene, except 3391/2017 virus, was obtained from Global Initiative on Sharing All Influenza Data (GISAID) EpiFlu database (Supplementary Table S1)^40^.

### Sequencing of the hemagglutinin gene

Reverse transcription-polymerase chain reaction and sequencing of the hemagglutinin gene of 3391/2017 virus were performed as we described previously^41, 42^. Briefly, viral RNA was extracted using QIAamp Viral RNA Mini Kit (Qiagen, Hilden, Germany), and cDNA was reverse transcribed with Uni12 primer (5′-AGCAAAAGCAGG-3′) and PrimeScript RT reagent Kit (Takara Bio Inc., Shiga, Japan). The cDNA was amplified by influenza H3 specific primers. PCR products were gel purified with Wizard SV Gel and PCR Clean-Up System (Promega, Wisconsin, USA) before sequencing with an ABI Prism 3700 DNA Analyzer (Applied Biosystems, Massachusetts, USA). Sequence fragments were assembled with Lasergene 7.1 (DNASTAR). The sequence has been deposited into NCBI GenBank (Accession number MG693001).

### Phylogenetic analysis

The phylogenetic tree of the hemagglutinin gene was constructed using the Maximum Likelihood method based on the Hasegawa–Kishino–Yano model using MEGA software package version 7.0. The bootstrap values from 1000 replicates were calculated to evaluate the reliability of the phylogenetic trees.

### Amino acid sequence comparison

Amino acid sequences of all Hong Kong influenza A(H3N2) viruses with collection date between 1 January 2014 to 13 December 2017 and deposited into GISAID EpiFlu database.

Bioedit version 7.0.9.0 was used for alignment and analysis of the amino acid residues.

### Statistical analysis

Statistical analysis was performed using SPSS 21.0 and GraphPad PRISM version 6.0. Comparison of geometric mean antibody titer was performed using log-transformed antibody titer with pair-sampled *t*-test. Fisher’s exact test was performed to compare categorical variables.

## Electronic supplementary material

Supplementary Table S1
