# Supplementary material for: Low population serum microneutralization antibody titer against the predominating influenza A(H3N2) N121K virus during the severe influenza summer peak of Hong Kong in 2017
Source: Emerg Microbes Infect. 2018 Mar 6;7:23. doi: 10.1038/s41426-018-0041-1 (PMC5841213; doi:10.1038/s41426-018-0041-1)
Supplement: Supplementary file 1 — Supplementary Table S1 [file 41426_2018_41_MOESM1_ESM.doc]

**Supplementary Table S1**. Details of influenza A(H3N2) hemagglutinin sequences obtained from GISAID EpiFlu^TM^ database that are used in the amino acid sequence and phylogenetic analysis in this study.

| **Segment ID** | **Segment** | **Country** | **Collection date** | **Isolate name** | **Originating Lab** | **Submitting Lab** |
| --- | --- | --- | --- | --- | --- | --- |
|  |  |  |  |  |  |  |
| EPI1073846 | HA | Hong Kong (SAR) | 2017-Jul-17 | A/Hong Kong/3820/2017 | Government Virus Unit | Crick Worldwide Influenza Centre |
| EPI1073832 | HA | Hong Kong (SAR) | 2017-Jul-04 | A/Hong Kong/3162/2017 | Government Virus Unit | Crick Worldwide Influenza Centre |
| EPI1060886 | HA | Hong Kong (SAR) | 2017-Jul-23 | A/Hong Kong/3855/2017 | Government Virus Unit | Crick Worldwide Influenza Centre |
| EPI1060884 | HA | Hong Kong (SAR) | 2017-Jul-21 | A/Hong Kong/3854/2017 | Government Virus Unit | Crick Worldwide Influenza Centre |
| EPI1060882 | HA | Hong Kong (SAR) | 2017-Jul-24 | A/Hong Kong/3853/2017 | Government Virus Unit | Crick Worldwide Influenza Centre |
| EPI1060880 | HA | Hong Kong (SAR) | 2017-Jul-20 | A/Hong Kong/3825/2017 | Government Virus Unit | Crick Worldwide Influenza Centre |
| EPI1060878 | HA | Hong Kong (SAR) | 2017-Jul-21 | A/Hong Kong/3824/2017 | Government Virus Unit | Crick Worldwide Influenza Centre |
| EPI1060876 | HA | Hong Kong (SAR) | 2017-Jul-18 | A/Hong Kong/3823/2017 | Government Virus Unit | Crick Worldwide Influenza Centre |
| EPI1060874 | HA | Hong Kong (SAR) | 2017-Jul-18 | A/Hong Kong/3821/2017 | Government Virus Unit | Crick Worldwide Influenza Centre |
| EPI1060872 | HA | Hong Kong (SAR) | 2017-Jul-15 | A/Hong Kong/3819/2017 | Government Virus Unit | Crick Worldwide Influenza Centre |
| EPI1060870 | HA | Hong Kong (SAR) | 2017-Jul-13 | A/Hong Kong/3815/2017 | Government Virus Unit | Crick Worldwide Influenza Centre |
| EPI1060868 | HA | Hong Kong (SAR) | 2017-Jul-20 | A/Hong Kong/3800/2017 | Government Virus Unit | Crick Worldwide Influenza Centre |
| EPI1060866 | HA | Hong Kong (SAR) | 2017-Jul-11 | A/Hong Kong/3730/2017 | Government Virus Unit | Crick Worldwide Influenza Centre |
| EPI1060864 | HA | Hong Kong (SAR) | 2017-Jul-08 | A/Hong Kong/3345/2017 | Government Virus Unit | Crick Worldwide Influenza Centre |
| EPI1060838 | HA | Hong Kong (SAR) | 2017-Jun-28 | A/Hong Kong/3043/2017 | Government Virus Unit | Crick Worldwide Influenza Centre |
| EPI1060834 | HA | Hong Kong (SAR) | 2017-Jun-04 | A/Hong Kong/2473/2017 | Government Virus Unit | Crick Worldwide Influenza Centre |
| EPI1057690 | HA | Hong Kong (SAR) | 2017-May-19 | A/Hong Kong/2261/2017 | Government Virus Unit | Crick Worldwide Influenza Centre |
| EPI1057688 | HA | Hong Kong (SAR) | 2017-May-19 | A/Hong Kong/2227/2017 | Government Virus Unit | Crick Worldwide Influenza Centre |
| EPI1043991 | HA | Hong Kong (SAR) | 2017-Jul-03 | A/Hong Kong/3163/2017 | Government Virus Unit | Crick Worldwide Influenza Centre |
| EPI1043989 | HA | Hong Kong (SAR) | 2017-Jul-04 | A/Hong Kong/3162/2017 | Government Virus Unit | Crick Worldwide Influenza Centre |
| EPI1043987 | HA | Hong Kong (SAR) | 2017-Jul-03 | A/Hong Kong/3161/2017 | Government Virus Unit | Crick Worldwide Influenza Centre |
| EPI1043985 | HA | Hong Kong (SAR) | 2017-Jul-04 | A/Hong Kong/3160/2017 | Government Virus Unit | Crick Worldwide Influenza Centre |
| EPI1043983 | HA | Hong Kong (SAR) | 2017-Jul-03 | A/Hong Kong/3159/2017 | Government Virus Unit | Crick Worldwide Influenza Centre |
| EPI1043981 | HA | Hong Kong (SAR) | 2017-Jul-02 | A/Hong Kong/3158/2017 | Government Virus Unit | Crick Worldwide Influenza Centre |
| EPI1043979 | HA | Hong Kong (SAR) | 2017-Jul-02 | A/Hong Kong/3157/2017 | Government Virus Unit | Crick Worldwide Influenza Centre |
| EPI1043977 | HA | Hong Kong (SAR) | 2017-Jul-02 | A/Hong Kong/3156/2017 | Government Virus Unit | Crick Worldwide Influenza Centre |
| EPI1043975 | HA | Hong Kong (SAR) | 2017-Jul-04 | A/Hong Kong/3141/2017 | Government Virus Unit | Crick Worldwide Influenza Centre |
| EPI1043973 | HA | Hong Kong (SAR) | 2017-Jul-03 | A/Hong Kong/3140/2017 | Government Virus Unit | Crick Worldwide Influenza Centre |
| EPI1043971 | HA | Hong Kong (SAR) | 2017-Jul-03 | A/Hong Kong/3139/2017 | Government Virus Unit | Crick Worldwide Influenza Centre |
| EPI1043969 | HA | Hong Kong (SAR) | 2017-Jul-03 | A/Hong Kong/3138/2017 | Government Virus Unit | Crick Worldwide Influenza Centre |
| EPI1043967 | HA | Hong Kong (SAR) | 2017-Jul-03 | A/Hong Kong/3137/2017 | Government Virus Unit | Crick Worldwide Influenza Centre |
| EPI1043965 | HA | Hong Kong (SAR) | 2017-Jul-02 | A/Hong Kong/3136/2017 | Government Virus Unit | Crick Worldwide Influenza Centre |
| EPI1043963 | HA | Hong Kong (SAR) | 2017-Jul-02 | A/Hong Kong/3135/2017 | Government Virus Unit | Crick Worldwide Influenza Centre |
| EPI1043961 | HA | Hong Kong (SAR) | 2017-Jul-03 | A/Hong Kong/3134/2017 | Government Virus Unit | Crick Worldwide Influenza Centre |
| EPI1043959 | HA | Hong Kong (SAR) | 2017-Jul-03 | A/Hong Kong/3132/2017 | Government Virus Unit | Crick Worldwide Influenza Centre |
| EPI1043957 | HA | Hong Kong (SAR) | 2017-Jul-02 | A/Hong Kong/3131/2017 | Government Virus Unit | Crick Worldwide Influenza Centre |
| EPI1043955 | HA | Hong Kong (SAR) | 2017-Jul-02 | A/Hong Kong/3130/2017 | Government Virus Unit | Crick Worldwide Influenza Centre |
| EPI1043953 | HA | Hong Kong (SAR) | 2017-Jul-03 | A/Hong Kong/3129/2017 | Government Virus Unit | Crick Worldwide Influenza Centre |
| EPI1043951 | HA | Hong Kong (SAR) | 2017-Jul-04 | A/Hong Kong/3128/2017 | Government Virus Unit | Crick Worldwide Influenza Centre |
| EPI1043949 | HA | Hong Kong (SAR) | 2017-Jul-02 | A/Hong Kong/3127/2017 | Government Virus Unit | Crick Worldwide Influenza Centre |
| EPI1043947 | HA | Hong Kong (SAR) | 2017-Jul-03 | A/Hong Kong/3126/2017 | Government Virus Unit | Crick Worldwide Influenza Centre |
| EPI1043945 | HA | Hong Kong (SAR) | 2017-Jul-02 | A/Hong Kong/3125/2017 | Government Virus Unit | Crick Worldwide Influenza Centre |
| EPI1043943 | HA | Hong Kong (SAR) | 2017-Jul-03 | A/Hong Kong/3124/2017 | Government Virus Unit | Crick Worldwide Influenza Centre |
| EPI1043941 | HA | Hong Kong (SAR) | 2017-Jul-03 | A/Hong Kong/3123/2017 | Government Virus Unit | Crick Worldwide Influenza Centre |
| EPI1043939 | HA | Hong Kong (SAR) | 2017-Jul-02 | A/Hong Kong/3122/2017 | Government Virus Unit | Crick Worldwide Influenza Centre |
| EPI1043937 | HA | Hong Kong (SAR) | 2017-Jul-03 | A/Hong Kong/3121/2017 | Government Virus Unit | Crick Worldwide Influenza Centre |
| EPI1043935 | HA | Hong Kong (SAR) | 2017-Jul-01 | A/Hong Kong/3119/2017 | Government Virus Unit | Crick Worldwide Influenza Centre |
| EPI1043933 | HA | Hong Kong (SAR) | 2017-Jun-30 | A/Hong Kong/3118/2017 | Government Virus Unit | Crick Worldwide Influenza Centre |
| EPI1043931 | HA | Hong Kong (SAR) | 2017-Jul-01 | A/Hong Kong/3117/2017 | Government Virus Unit | Crick Worldwide Influenza Centre |
| EPI1043929 | HA | Hong Kong (SAR) | 2017-Jun-30 | A/Hong Kong/3116/2017 | Government Virus Unit | Crick Worldwide Influenza Centre |
| EPI1043927 | HA | Hong Kong (SAR) | 2017-Jul-01 | A/Hong Kong/3115/2017 | Government Virus Unit | Crick Worldwide Influenza Centre |
| EPI1043925 | HA | Hong Kong (SAR) | 2017-Jul-01 | A/Hong Kong/3114/2017 | Government Virus Unit | Crick Worldwide Influenza Centre |
| EPI1043923 | HA | Hong Kong (SAR) | 2017-Jul-03 | A/Hong Kong/3113/2017 | Government Virus Unit | Crick Worldwide Influenza Centre |
| EPI1043921 | HA | Hong Kong (SAR) | 2017-Jul-02 | A/Hong Kong/3112/2017 | Government Virus Unit | Crick Worldwide Influenza Centre |
| EPI1043919 | HA | Hong Kong (SAR) | 2017-Jul-01 | A/Hong Kong/3111/2017 | Government Virus Unit | Crick Worldwide Influenza Centre |
| EPI1043917 | HA | Hong Kong (SAR) | 2017-Jul-03 | A/Hong Kong/3110/2017 | Government Virus Unit | Crick Worldwide Influenza Centre |
| EPI1043915 | HA | Hong Kong (SAR) | 2017-Jul-02 | A/Hong Kong/3108/2017 | Government Virus Unit | Crick Worldwide Influenza Centre |
| EPI1043913 | HA | Hong Kong (SAR) | 2017-Jul-01 | A/Hong Kong/3107/2017 | Government Virus Unit | Crick Worldwide Influenza Centre |
| EPI1043911 | HA | Hong Kong (SAR) | 2017-Jul-02 | A/Hong Kong/3106/2017 | Government Virus Unit | Crick Worldwide Influenza Centre |
| EPI1043909 | HA | Hong Kong (SAR) | 2017-Jun-29 | A/Hong Kong/3105/2017 | Government Virus Unit | Crick Worldwide Influenza Centre |
| EPI1043907 | HA | Hong Kong (SAR) | 2017-Jun-29 | A/Hong Kong/3104/2017 | Government Virus Unit | Crick Worldwide Influenza Centre |
| EPI1043905 | HA | Hong Kong (SAR) | 2017-Jun-29 | A/Hong Kong/3102/2017 | Government Virus Unit | Crick Worldwide Influenza Centre |
| EPI1043903 | HA | Hong Kong (SAR) | 2017-Jun-28 | A/Hong Kong/3101/2017 | Government Virus Unit | Crick Worldwide Influenza Centre |
| EPI1043901 | HA | Hong Kong (SAR) | 2017-Jul-01 | A/Hong Kong/3099/2017 | Government Virus Unit | Crick Worldwide Influenza Centre |
| EPI1043899 | HA | Hong Kong (SAR) | 2017-Jul-01 | A/Hong Kong/3098/2017 | Government Virus Unit | Crick Worldwide Influenza Centre |
| EPI1043897 | HA | Hong Kong (SAR) | 2017-Jul-01 | A/Hong Kong/3097/2017 | Government Virus Unit | Crick Worldwide Influenza Centre |
| EPI1043895 | HA | Hong Kong (SAR) | 2017-Jul-01 | A/Hong Kong/3096/2017 | Government Virus Unit | Crick Worldwide Influenza Centre |
| EPI1043893 | HA | Hong Kong (SAR) | 2017-Jul-01 | A/Hong Kong/3095/2017 | Government Virus Unit | Crick Worldwide Influenza Centre |
| EPI1043891 | HA | Hong Kong (SAR) | 2017-Jul-01 | A/Hong Kong/3094/2017 | Government Virus Unit | Crick Worldwide Influenza Centre |
| EPI1043889 | HA | Hong Kong (SAR) | 2017-Jul-01 | A/Hong Kong/3093/2017 | Government Virus Unit | Crick Worldwide Influenza Centre |
| EPI1043887 | HA | Hong Kong (SAR) | 2017-Jul-01 | A/Hong Kong/3092/2017 | Government Virus Unit | Crick Worldwide Influenza Centre |
| EPI1043885 | HA | Hong Kong (SAR) | 2017-Jun-30 | A/Hong Kong/3091/2017 | Government Virus Unit | Crick Worldwide Influenza Centre |
| EPI1043883 | HA | Hong Kong (SAR) | 2017-Jul-03 | A/Hong Kong/3090/2017 | Government Virus Unit | Crick Worldwide Influenza Centre |
| EPI1043881 | HA | Hong Kong (SAR) | 2017-Jul-01 | A/Hong Kong/3089/2017 | Government Virus Unit | Crick Worldwide Influenza Centre |
| EPI1043879 | HA | Hong Kong (SAR) | 2017-Jul-02 | A/Hong Kong/3088/2017 | Government Virus Unit | Crick Worldwide Influenza Centre |
| EPI1043877 | HA | Hong Kong (SAR) | 2017-Jul-01 | A/Hong Kong/3086/2017 | Government Virus Unit | Crick Worldwide Influenza Centre |
| EPI1043875 | HA | Hong Kong (SAR) | 2017-Jul-02 | A/Hong Kong/3085/2017 | Government Virus Unit | Crick Worldwide Influenza Centre |
| EPI1043873 | HA | Hong Kong (SAR) | 2017-Jun-30 | A/Hong Kong/3084/2017 | Government Virus Unit | Crick Worldwide Influenza Centre |
| EPI1043871 | HA | Hong Kong (SAR) | 2017-Jun-30 | A/Hong Kong/3083/2017 | Government Virus Unit | Crick Worldwide Influenza Centre |
| EPI1043869 | HA | Hong Kong (SAR) | 2017-Jun-29 | A/Hong Kong/3082/2017 | Government Virus Unit | Crick Worldwide Influenza Centre |
| EPI1043867 | HA | Hong Kong (SAR) | 2017-Jun-30 | A/Hong Kong/3081/2017 | Government Virus Unit | Crick Worldwide Influenza Centre |
| EPI1043865 | HA | Hong Kong (SAR) | 2017-Jun-29 | A/Hong Kong/3080/2017 | Government Virus Unit | Crick Worldwide Influenza Centre |
| EPI1043863 | HA | Hong Kong (SAR) | 2017-Jun-28 | A/Hong Kong/3079/2017 | Government Virus Unit | Crick Worldwide Influenza Centre |
| EPI1043861 | HA | Hong Kong (SAR) | 2017-Jun-29 | A/Hong Kong/3077/2017 | Government Virus Unit | Crick Worldwide Influenza Centre |
| EPI1043859 | HA | Hong Kong (SAR) | 2017-Jun-29 | A/Hong Kong/3076/2017 | Government Virus Unit | Crick Worldwide Influenza Centre |
| EPI1043857 | HA | Hong Kong (SAR) | 2017-Jun-29 | A/Hong Kong/3075/2017 | Government Virus Unit | Crick Worldwide Influenza Centre |
| EPI1043855 | HA | Hong Kong (SAR) | 2017-Jun-29 | A/Hong Kong/3074/2017 | Government Virus Unit | Crick Worldwide Influenza Centre |
| EPI1043853 | HA | Hong Kong (SAR) | 2017-Jun-29 | A/Hong Kong/3073/2017 | Government Virus Unit | Crick Worldwide Influenza Centre |
| EPI1043851 | HA | Hong Kong (SAR) | 2017-Jun-29 | A/Hong Kong/3072/2017 | Government Virus Unit | Crick Worldwide Influenza Centre |
| EPI1043849 | HA | Hong Kong (SAR) | 2017-Jun-29 | A/Hong Kong/3071/2017 | Government Virus Unit | Crick Worldwide Influenza Centre |
| EPI1043847 | HA | Hong Kong (SAR) | 2017-Jun-29 | A/Hong Kong/3070/2017 | Government Virus Unit | Crick Worldwide Influenza Centre |
| EPI1036347 | HA | Hong Kong (SAR) | 2017-Jun-29 | A/Hong Kong/3068/2017 | Government Virus Unit | Crick Worldwide Influenza Centre |
| EPI1036345 | HA | Hong Kong (SAR) | 2017-Jun-29 | A/Hong Kong/3066/2017 | Government Virus Unit | Crick Worldwide Influenza Centre |
| EPI1036343 | HA | Hong Kong (SAR) | 2017-Jun-29 | A/Hong Kong/3065/2017 | Government Virus Unit | Crick Worldwide Influenza Centre |
| EPI1036341 | HA | Hong Kong (SAR) | 2017-Jun-29 | A/Hong Kong/3064/2017 | Government Virus Unit | Crick Worldwide Influenza Centre |
| EPI1036339 | HA | Hong Kong (SAR) | 2017-Jun-29 | A/Hong Kong/3062/2017 | Government Virus Unit | Crick Worldwide Influenza Centre |
| EPI1036337 | HA | Hong Kong (SAR) | 2017-Jun-29 | A/Hong Kong/3061/2017 | Government Virus Unit | Crick Worldwide Influenza Centre |
| EPI1036335 | HA | Hong Kong (SAR) | 2017-Jun-29 | A/Hong Kong/3060/2017 | Government Virus Unit | Crick Worldwide Influenza Centre |
| EPI1036333 | HA | Hong Kong (SAR) | 2017-Jun-29 | A/Hong Kong/3059/2017 | Government Virus Unit | Crick Worldwide Influenza Centre |
| EPI1036331 | HA | Hong Kong (SAR) | 2017-Jun-29 | A/Hong Kong/3056/2017 | Government Virus Unit | Crick Worldwide Influenza Centre |
| EPI1036329 | HA | Hong Kong (SAR) | 2017-Jun-28 | A/Hong Kong/3055/2017 | Government Virus Unit | Crick Worldwide Influenza Centre |
| EPI1036327 | HA | Hong Kong (SAR) | 2017-Jun-27 | A/Hong Kong/3054/2017 | Government Virus Unit | Crick Worldwide Influenza Centre |
| EPI1036325 | HA | Hong Kong (SAR) | 2017-Jun-27 | A/Hong Kong/3053/2017 | Government Virus Unit | Crick Worldwide Influenza Centre |
| EPI1036323 | HA | Hong Kong (SAR) | 2017-Jun-28 | A/Hong Kong/3052/2017 | Government Virus Unit | Crick Worldwide Influenza Centre |
| EPI1036321 | HA | Hong Kong (SAR) | 2017-Jun-27 | A/Hong Kong/3051/2017 | Government Virus Unit | Crick Worldwide Influenza Centre |
| EPI1036319 | HA | Hong Kong (SAR) | 2017-Jun-30 | A/Hong Kong/3046/2017 | Government Virus Unit | Crick Worldwide Influenza Centre |
| EPI1036317 | HA | Hong Kong (SAR) | 2017-Jun-30 | A/Hong Kong/3045/2017 | Government Virus Unit | Crick Worldwide Influenza Centre |
| EPI1036315 | HA | Hong Kong (SAR) | 2017-Jun-27 | A/Hong Kong/3044/2017 | Government Virus Unit | Crick Worldwide Influenza Centre |
| EPI1036313 | HA | Hong Kong (SAR) | 2017-Jun-27 | A/Hong Kong/3042/2017 | Government Virus Unit | Crick Worldwide Influenza Centre |
| EPI1021224 | HA | Hong Kong (SAR) | 2017-May-23 | A/Hong Kong/2293/2017 | Government Virus Unit | Crick Worldwide Influenza Centre |
| EPI1021222 | HA | Hong Kong (SAR) | 2017-May-22 | A/Hong Kong/2292/2017 | Government Virus Unit | Crick Worldwide Influenza Centre |
| EPI1021220 | HA | Hong Kong (SAR) | 2017-May-23 | A/Hong Kong/2291/2017 | Government Virus Unit | Crick Worldwide Influenza Centre |
| EPI1021218 | HA | Hong Kong (SAR) | 2017-May-23 | A/Hong Kong/2290/2017 | Government Virus Unit | Crick Worldwide Influenza Centre |
| EPI1021216 | HA | Hong Kong (SAR) | 2017-May-23 | A/Hong Kong/2289/2017 | Government Virus Unit | Crick Worldwide Influenza Centre |
| EPI1021214 | HA | Hong Kong (SAR) | 2017-May-23 | A/Hong Kong/2288/2017 | Government Virus Unit | Crick Worldwide Influenza Centre |
| EPI1021212 | HA | Hong Kong (SAR) | 2017-May-24 | A/Hong Kong/2287/2017 | Government Virus Unit | Crick Worldwide Influenza Centre |
| EPI1021210 | HA | Hong Kong (SAR) | 2017-May-23 | A/Hong Kong/2286/2017 | Government Virus Unit | Crick Worldwide Influenza Centre |
| EPI1021208 | HA | Hong Kong (SAR) | 2017-May-23 | A/Hong Kong/2285/2017 | Government Virus Unit | Crick Worldwide Influenza Centre |
| EPI1021206 | HA | Hong Kong (SAR) | 2017-May-23 | A/Hong Kong/2284/2017 | Government Virus Unit | Crick Worldwide Influenza Centre |
| EPI1021204 | HA | Hong Kong (SAR) | 2017-May-23 | A/Hong Kong/2283/2017 | Government Virus Unit | Crick Worldwide Influenza Centre |
| EPI1021202 | HA | Hong Kong (SAR) | 2017-May-20 | A/Hong Kong/2281/2017 | Government Virus Unit | Crick Worldwide Influenza Centre |
| EPI1021200 | HA | Hong Kong (SAR) | 2017-May-20 | A/Hong Kong/2280/2017 | Government Virus Unit | Crick Worldwide Influenza Centre |
| EPI1021198 | HA | Hong Kong (SAR) | 2017-May-21 | A/Hong Kong/2279/2017 | Government Virus Unit | Crick Worldwide Influenza Centre |
| EPI1021196 | HA | Hong Kong (SAR) | 2017-May-19 | A/Hong Kong/2277/2017 | Government Virus Unit | Crick Worldwide Influenza Centre |
| EPI1021194 | HA | Hong Kong (SAR) | 2017-May-19 | A/Hong Kong/2276/2017 | Government Virus Unit | Crick Worldwide Influenza Centre |
| EPI1021192 | HA | Hong Kong (SAR) | 2017-May-20 | A/Hong Kong/2275/2017 | Government Virus Unit | Crick Worldwide Influenza Centre |
| EPI1021190 | HA | Hong Kong (SAR) | 2017-May-20 | A/Hong Kong/2274/2017 | Government Virus Unit | Crick Worldwide Influenza Centre |
| EPI1021188 | HA | Hong Kong (SAR) | 2017-May-21 | A/Hong Kong/2273/2017 | Government Virus Unit | Crick Worldwide Influenza Centre |
| EPI1021186 | HA | Hong Kong (SAR) | 2017-May-20 | A/Hong Kong/2272/2017 | Government Virus Unit | Crick Worldwide Influenza Centre |
| EPI1021184 | HA | Hong Kong (SAR) | 2017-May-21 | A/Hong Kong/2271/2017 | Government Virus Unit | Crick Worldwide Influenza Centre |
| EPI1021182 | HA | Hong Kong (SAR) | 2017-May-20 | A/Hong Kong/2270/2017 | Government Virus Unit | Crick Worldwide Influenza Centre |
| EPI1021180 | HA | Hong Kong (SAR) | 2017-May-20 | A/Hong Kong/2269/2017 | Government Virus Unit | Crick Worldwide Influenza Centre |
| EPI1021178 | HA | Hong Kong (SAR) | 2017-May-19 | A/Hong Kong/2267/2017 | Government Virus Unit | Crick Worldwide Influenza Centre |
| EPI1021176 | HA | Hong Kong (SAR) | 2017-May-20 | A/Hong Kong/2266/2017 | Government Virus Unit | Crick Worldwide Influenza Centre |
| EPI1021174 | HA | Hong Kong (SAR) | 2017-May-20 | A/Hong Kong/2265/2017 | Government Virus Unit | Crick Worldwide Influenza Centre |
| EPI1021172 | HA | Hong Kong (SAR) | 2017-May-19 | A/Hong Kong/2263/2017 | Government Virus Unit | Crick Worldwide Influenza Centre |
| EPI1021170 | HA | Hong Kong (SAR) | 2017-May-19 | A/Hong Kong/2262/2017 | Government Virus Unit | Crick Worldwide Influenza Centre |
| EPI1021168 | HA | Hong Kong (SAR) | 2017-May-19 | A/Hong Kong/2260/2017 | Government Virus Unit | Crick Worldwide Influenza Centre |
| EPI1021166 | HA | Hong Kong (SAR) | 2017-May-19 | A/Hong Kong/2259/2017 | Government Virus Unit | Crick Worldwide Influenza Centre |
| EPI1021164 | HA | Hong Kong (SAR) | 2017-May-19 | A/Hong Kong/2258/2017 | Government Virus Unit | Crick Worldwide Influenza Centre |
| EPI1021162 | HA | Hong Kong (SAR) | 2017-May-19 | A/Hong Kong/2257/2017 | Government Virus Unit | Crick Worldwide Influenza Centre |
| EPI1021160 | HA | Hong Kong (SAR) | 2017-May-19 | A/Hong Kong/2256/2017 | Government Virus Unit | Crick Worldwide Influenza Centre |
| EPI1021158 | HA | Hong Kong (SAR) | 2017-May-19 | A/Hong Kong/2255/2017 | Government Virus Unit | Crick Worldwide Influenza Centre |
| EPI1021156 | HA | Hong Kong (SAR) | 2017-May-19 | A/Hong Kong/2230/2017 | Government Virus Unit | Crick Worldwide Influenza Centre |
| EPI1021154 | HA | Hong Kong (SAR) | 2017-May-19 | A/Hong Kong/2229/2017 | Government Virus Unit | Crick Worldwide Influenza Centre |
| EPI1021152 | HA | Hong Kong (SAR) | 2017-May-20 | A/Hong Kong/2228/2017 | Government Virus Unit | Crick Worldwide Influenza Centre |
| EPI1021150 | HA | Hong Kong (SAR) | 2017-May-20 | A/Hong Kong/2226/2017 | Government Virus Unit | Crick Worldwide Influenza Centre |
| EPI943228 | HA | Hong Kong (SAR) | 2017-Jan-07 | A/Hong Kong/240/2017 | Government Virus Unit | Crick Worldwide Influenza Centre |
| EPI943226 | HA | Hong Kong (SAR) | 2017-Jan-04 | A/Hong Kong/120/2017 | Government Virus Unit | Crick Worldwide Influenza Centre |
| EPI943224 | HA | Hong Kong (SAR) | 2017-Jan-04 | A/Hong Kong/119/2017 | Government Virus Unit | Crick Worldwide Influenza Centre |
| EPI1107291 | HA | Hong Kong (SAR) | 2017-Oct-22 | A/Hong Kong/4829/2017 | Government Virus Unit | Centers for Disease Control and Prevention |
| EPI1107283 | HA | Hong Kong (SAR) | 2017-Oct-23 | A/Hong Kong/4828/2017 | Government Virus Unit | Centers for Disease Control and Prevention |
| EPI1107123 | HA | Hong Kong (SAR) | 2017-Oct-20 | A/Hong Kong/4825/2017 | Government Virus Unit | Centers for Disease Control and Prevention |
| EPI1107099 | HA | Hong Kong (SAR) | 2017-Oct-22 | A/Hong Kong/4824/2017 | Government Virus Unit | Centers for Disease Control and Prevention |
| EPI1107091 | HA | Hong Kong (SAR) | 2017-Oct-21 | A/Hong Kong/4823/2017 | Government Virus Unit | Centers for Disease Control and Prevention |
| EPI1107075 | HA | Hong Kong (SAR) | 2017-Oct-23 | A/Hong Kong/4819/2017 | Government Virus Unit | Centers for Disease Control and Prevention |
| EPI1107060 | HA | Hong Kong (SAR) | 2017-Oct-22 | A/Hong Kong/4818/2017 | Government Virus Unit | Centers for Disease Control and Prevention |
| EPI1055926 | HA | Hong Kong (SAR) | 2017-Jul-20 | A/Hong Kong/3630/2017 | Government Virus Unit | Centers for Disease Control and Prevention |
| EPI1048754 | HA | Hong Kong (SAR) | 2017-Jul-18 | A/Hong Kong/3629/2017 | Government Virus Unit | Centers for Disease Control and Prevention |
| EPI1048738 | HA | Hong Kong (SAR) | 2017-Jul-14 | A/Hong Kong/3563/2017 | Government Virus Unit | Centers for Disease Control and Prevention |
| EPI1048723 | HA | Hong Kong (SAR) | 2017-Jul-15 | A/Hong Kong/3596/2017 | Government Virus Unit | Centers for Disease Control and Prevention |
| EPI1048708 | HA | Hong Kong (SAR) | 2017-Jul-16 | A/Hong Kong/3595/2017 | Government Virus Unit | Centers for Disease Control and Prevention |
| EPI1048692 | HA | Hong Kong (SAR) | 2017-Jul-16 | A/Hong Kong/3578/2017 | Government Virus Unit | Centers for Disease Control and Prevention |
| EPI1048675 | HA | Hong Kong (SAR) | 2017-Jul-14 | A/Hong Kong/3572/2017 | Government Virus Unit | Centers for Disease Control and Prevention |
| EPI1048661 | HA | Hong Kong (SAR) | 2017-Jul-18 | A/Hong Kong/3571/2017 | Government Virus Unit | Centers for Disease Control and Prevention |
| EPI1048642 | HA | Hong Kong (SAR) | 2017-Jul-19 | A/Hong Kong/3570/2017 | Government Virus Unit | Centers for Disease Control and Prevention |
| EPI1048629 | HA | Hong Kong (SAR) | 2017-Jul-15 | A/Hong Kong/3569/2017 | Government Virus Unit | Centers for Disease Control and Prevention |
| EPI1048614 | HA | Hong Kong (SAR) | 2017-Jul-15 | A/Hong Kong/3568/2017 | Government Virus Unit | Centers for Disease Control and Prevention |
| EPI1048599 | HA | Hong Kong (SAR) | 2017-Jul-14 | A/Hong Kong/3566/2017 | Government Virus Unit | Centers for Disease Control and Prevention |
| EPI1048584 | HA | Hong Kong (SAR) | 2017-Jul-14 | A/Hong Kong/3565/2017 | Government Virus Unit | Centers for Disease Control and Prevention |
| EPI1048567 | HA | Hong Kong (SAR) | 2017-Jul-11 | A/Hong Kong/3512/2017 | Government Virus Unit | Centers for Disease Control and Prevention |
| EPI1048554 | HA | Hong Kong (SAR) | 2017-Jul-16 | A/Hong Kong/3552/2017 | Government Virus Unit | Centers for Disease Control and Prevention |
| EPI1048537 | HA | Hong Kong (SAR) | 2017-Jul-16 | A/Hong Kong/3551/2017 | Government Virus Unit | Centers for Disease Control and Prevention |
| EPI1048521 | HA | Hong Kong (SAR) | 2017-Jul-16 | A/Hong Kong/3550/2017 | Government Virus Unit | Centers for Disease Control and Prevention |
| EPI1048505 | HA | Hong Kong (SAR) | 2017-Jul-16 | A/Hong Kong/3549/2017 | Government Virus Unit | Centers for Disease Control and Prevention |
| EPI1048488 | HA | Hong Kong (SAR) | 2017-Jul-16 | A/Hong Kong/3548/2017 | Government Virus Unit | Centers for Disease Control and Prevention |
| EPI1048471 | HA | Hong Kong (SAR) | 2017-Jul-16 | A/Hong Kong/3547/2017 | Government Virus Unit | Centers for Disease Control and Prevention |
| EPI1048456 | HA | Hong Kong (SAR) | 2017-Jul-16 | A/Hong Kong/3557/2017 | Government Virus Unit | Centers for Disease Control and Prevention |
| EPI1048439 | HA | Hong Kong (SAR) | 2017-Jul-15 | A/Hong Kong/3556/2017 | Government Virus Unit | Centers for Disease Control and Prevention |
| EPI1048420 | HA | Hong Kong (SAR) | 2017-Jul-15 | A/Hong Kong/3555/2017 | Government Virus Unit | Centers for Disease Control and Prevention |
| EPI1048403 | HA | Hong Kong (SAR) | 2017-Jul-15 | A/Hong Kong/3554/2017 | Government Virus Unit | Centers for Disease Control and Prevention |
| EPI1048387 | HA | Hong Kong (SAR) | 2017-Jul-17 | A/Hong Kong/3553/2017 | Government Virus Unit | Centers for Disease Control and Prevention |
| EPI960548 | HA | Hong Kong (SAR) | 2017-Jan-08 | A/Hong Kong/235/2017 | Government Virus Unit | Centers for Disease Control and Prevention |
| EPI918300 | HA | Hong Kong (SAR) | 2017-Jan-08 | A/Hong Kong/247/2017 | Government Virus Unit | Centers for Disease Control and Prevention |
| EPI918292 | HA | Hong Kong (SAR) | 2017-Jan-08 | A/Hong Kong/237/2017 | Government Virus Unit | Centers for Disease Control and Prevention |
| EPI918276 | HA | Hong Kong (SAR) | 2017-Jan-04 | A/Hong Kong/123/2017 | Government Virus Unit | Centers for Disease Control and Prevention |
| EPI918268 | HA | Hong Kong (SAR) | 2017-Jan-04 | A/Hong Kong/122/2017 | Government Virus Unit | Centers for Disease Control and Prevention |
| EPI918260 | HA | Hong Kong (SAR) | 2017-Jan-03 | A/Hong Kong/121/2017 | Government Virus Unit | Centers for Disease Control and Prevention |
| EPI918252 | HA | Hong Kong (SAR) | 2017-Jan-02 | A/Hong Kong/110/2017 | Government Virus Unit | Centers for Disease Control and Prevention |
| EPI780183 | HA | Singapore | 2016-Jun-14 | A/Singapore/INFIMH-16-0019/2016 | Ministry of Health, Singapore | Ministry of Health, Singapore |
| EPI539576 | HA | Hong Kong (SAR) | 2014-Feb-26 | A/Hong Kong/4801/2014 | Government Virus Unit | National Institute for Medical Research |
| EPI543763 | HA | Switzerland | 2013-Dec-06 | A/Switzerland/9715293/2013 | National Institute for Medical Research | National Institute of Infectious Diseases (NIID) |
| EPI556816 | HA | United States | 2012-Apr-15 | A/Texas/50/2012 | Texas Department of State Health Services-Laboratory Services | Centers for Disease Control and Prevention |
| EPI182941 | HA | Australia | 2009-Apr-07 | A/Perth/16/2009 | Pathwest QE II Medical Centre | WHO Collaborating Centre for Reference and Research on Influenza |
| EPI914402 | HA | United Kingdom | 2017-Jan-05 | A/England/1/2017 | Microbiology Services Colindale, Public Health England | Microbiology Services Colindale, Public Health England |
| EPI914098 | HA | United Kingdom | 2016-Dec-12 | A/England/65040088/2016 | Microbiology Services Colindale, Public Health England | Microbiology Services Colindale, Public Health England |
| EPI914210 | HA | United Kingdom | 2016-Dec-23 | A/England/65180254/2016 | Microbiology Services Colindale, Public Health England | Microbiology Services Colindale, Public Health England |
| EPI914290 | HA | United Kingdom | 2016-Dec-30 | A/England/65280021/2016 | Microbiology Services Colindale, Public Health England | Microbiology Services Colindale, Public Health England |
| EPI914314 | HA | United Kingdom | 2016-Dec-30 | A/England/70120008/2016 | Microbiology Services Colindale, Public Health England | Microbiology Services Colindale, Public Health England |
| EPI914330 | HA | United Kingdom | 2017-Jan-03 | A/England/70120033/2017 | Microbiology Services Colindale, Public Health England | Microbiology Services Colindale, Public Health England |
| EPI914354 | HA | United Kingdom | 2016-Dec-30 | A/England/70140008/2016 | Microbiology Services Colindale, Public Health England | Microbiology Services Colindale, Public Health England |
| EPI914362 | HA | United Kingdom | 2017-Jan-03 | A/England/70160064/2017 | Microbiology Services Colindale, Public Health England | Microbiology Services Colindale, Public Health England |
| EPI914370 | HA | United Kingdom | 2017-Jan-03 | A/England/70160070/2017 | Microbiology Services Colindale, Public Health England | Microbiology Services Colindale, Public Health England |
| EPI914410 | HA | United Kingdom | 2017-Jan-05 | A/England/70180012/2017 | Microbiology Services Colindale, Public Health England | Microbiology Services Colindale, Public Health England |
| EPI914795 | HA | United Kingdom | 2017-Jan-04 | A/England/70180024/2017 | Microbiology Services Colindale, Public Health England | Microbiology Services Colindale, Public Health England |
| EPI914434 | HA | United Kingdom | 2017-Jan-05 | A/England/70200021/2017 | Microbiology Services Colindale, Public Health England | Microbiology Services Colindale, Public Health England |
| EPI914458 | HA | United Kingdom | 2017-Jan-06 | A/England/70220042/2017 | Microbiology Services Colindale, Public Health England | Microbiology Services Colindale, Public Health England |
| EPI879344 | HA | Denmark | 2016-Nov-22 | A/Denmark/63/2016 | Statens Serum Institute | Statens Serum Institute |
| EPI879347 | HA | Denmark | 2016-Dec-06 | A/Denmark/72/2016 | Statens Serum Institute | Statens Serum Institute |
| EPI879366 | HA | Denmark | 2016-Dec-01 | A/Denmark/66/2016 | Statens Serum Institute | Statens Serum Institute |
| EPI879374 | HA | Denmark | 2016-Dec-18 | A/Denmark/101/2016 | Statens Serum Institute | Statens Serum Institute |
| EPI879375 | HA | Denmark | 2016-Dec-18 | A/Denmark/102/2016 | Statens Serum Institute | Statens Serum Institute |
| EPI879380 | HA | Denmark | 2016-Dec-16 | A/Denmark/107/2016 | Statens Serum Institute | Statens Serum Institute |
| EPI879382 | HA | Denmark | 2016-Dec-16 | A/Denmark/108/2016 | Statens Serum Institute | Statens Serum Institute |
| EPI913705 | HA | Denmark | 2016-Nov-17 | A/Denmark/59/2016 | Statens Serum Institute | Crick Worldwide Influenza Centre |
| EPI913711 | HA | Denmark | 2016-Dec-06 | A/Denmark/70/2016 | Statens Serum Institute | Crick Worldwide Influenza Centre |
| EPI829341 | HA | Norway | 2016-Jun-13 | A/Norway/3806/2016 | WHO National Influenza Centre | Crick Worldwide Influenza Centre |
| EPI836523 | HA | Hong Kong (SAR) | 2016-Aug-02 | A/Hong Kong/2398/2016 | Government Virus Unit | Centers for Disease Control and Prevention |
| EPI858889 | HA | Hong Kong (SAR) | 2016-Aug-08 | A/Hong Kong/2387/2016 | Government Virus Unit | Crick Worldwide Influenza Centre |
| EPI836515 | HA | Hong Kong (SAR) | 2016-Aug-09 | A/Hong Kong/2391/2016 | Government Virus Unit | Centers for Disease Control and Prevention |
| EPI836507 | HA | Hong Kong (SAR) | 2016-Aug-09 | A/Hong Kong/2389/2016 | Government Virus Unit | Centers for Disease Control and Prevention |
| EPI699738 | HA | Hong Kong (SAR) | 2016-Jan-01 | A/Hong Kong/97/2016 | Government Virus Unit | Crick Worldwide Influenza Centre |
| EPI718988 | HA | Hong Kong (SAR) | 2016-Jan-02 | A/Hong Kong/98/2016 | Government Virus Unit | Centers for Disease Control and Prevention |
| EPI858887 | HA | Hong Kong (SAR) | 2016-Jul-28 | A/Hong Kong/2271/2016 | Government Virus Unit | Crick Worldwide Influenza Centre |
| EPI858885 | HA | Hong Kong (SAR) | 2016-Jul-28 | A/Hong Kong/2262/2016 | Government Virus Unit | Crick Worldwide Influenza Centre |
| EPI836499 | HA | Hong Kong (SAR) | 2016-Jul-29 | A/Hong Kong/2302/2016 | Government Virus Unit | Centers for Disease Control and Prevention |
| EPI836491 | HA | Hong Kong (SAR) | 2016-Jul-30 | A/Hong Kong/2298/2016 | Government Virus Unit | Centers for Disease Control and Prevention |
| EPI814009 | HA | Hong Kong (SAR) | 2016-May-14 | A/Hong Kong/1706/2016 | Government Virus Unit | Centers for Disease Control and Prevention |
| EPI814001 | HA | Hong Kong (SAR) | 2016-May-14 | A/Hong Kong/1705/2016 | Government Virus Unit | Centers for Disease Control and Prevention |
| EPI813993 | HA | Hong Kong (SAR) | 2016-May-15 | A/Hong Kong/1704/2016 | Government Virus Unit | Centers for Disease Control and Prevention |
| EPI730943 | HA | Hong Kong (SAR) | 2015-Dec-27 | A/Hong Kong/40/2016 | Government Virus Unit | Centers for Disease Control and Prevention |
| EPI718972 | HA | Hong Kong (SAR) | 2015-Dec-29 | A/Hong Kong/39/2016 | Government Virus Unit | Centers for Disease Control and Prevention |
| EPI699736 | HA | Hong Kong (SAR) | 2015-Dec-30 | A/Hong Kong/50/2016 | Government Virus Unit | Crick Worldwide Influenza Centre |
| EPI574615 | HA | Hong Kong (SAR) | 2015-Jan-24 | A/Hong Kong/8112865/2015 | Government Virus Unit | National Institute for Medical Research |
| EPI574613 | HA | Hong Kong (SAR) | 2015-Jan-24 | A/Hong Kong/8112788/2015 | Government Virus Unit | National Institute for Medical Research |
| EPI574611 | HA | Hong Kong (SAR) | 2015-Jan-24 | A/Hong Kong/8112349/2015 | Government Virus Unit | National Institute for Medical Research |
| EPI630729 | HA | Hong Kong (SAR) | 2015-Jun-09 | A/Hong Kong/11706/2015 | Government Virus Unit | Crick Worldwide Influenza Centre |
| EPI630733 | HA | Hong Kong (SAR) | 2015-Jun-09 | A/Hong Kong/11743/2015 | Government Virus Unit | Crick Worldwide Influenza Centre |
| EPI631563 | HA | Hong Kong (SAR) | 2015-Jun-09 | A/Hong Kong/11709/2015 | Government Virus Unit | Centers for Disease Control and Prevention |
| EPI631556 | HA | Hong Kong (SAR) | 2015-Jun-10 | A/Hong Kong/11742/2015 | Government Virus Unit | Centers for Disease Control and Prevention |
| EPI631549 | HA | Hong Kong (SAR) | 2015-Jun-10 | A/Hong Kong/11741/2015 | Government Virus Unit | Centers for Disease Control and Prevention |
| EPI630731 | HA | Hong Kong (SAR) | 2015-Jun-10 | A/Hong Kong/11713/2015 | Government Virus Unit | Crick Worldwide Influenza Centre |
| EPI679454 | HA | Hong Kong (SAR) | 2015-Oct-09 | A/Hong Kong/15590/2015 | Government Virus Unit | Centers for Disease Control and Prevention |
| EPI679463 | HA | Hong Kong (SAR) | 2015-Oct-14 | A/Hong Kong/15607/2015 | Government Virus Unit | Centers for Disease Control and Prevention |
| EPI679471 | HA | Hong Kong (SAR) | 2015-Oct-17 | A/Hong Kong/15609/2015 | Government Virus Unit | Centers for Disease Control and Prevention |
| EPI679476 | HA | Hong Kong (SAR) | 2015-Oct-19 | A/Hong Kong/15611/2015 | Government Virus Unit | Centers for Disease Control and Prevention |
| EPI539802 | HA | Hong Kong (SAR) | 2014-Apr-04 | A/Hong Kong/5578/2014 | Government Virus Unit | National Institute for Medical Research |
| EPI551860 | HA | Hong Kong (SAR) | 2014-Apr-05 | A/Hong Kong/5576/2014 | Government Virus Unit | National Institute for Medical Research |
| EPI539804 | HA | Hong Kong (SAR) | 2014-Apr-21 | A/Hong Kong/5695/2014 | Government Virus Unit | National Institute for Medical Research |
| EPI539580 | HA | Hong Kong (SAR) | 2014-Apr-29 | A/Hong Kong/5739/2014 | Government Virus Unit | National Institute for Medical Research |
| EPI543726 | HA | Hong Kong (SAR) | 2014-Apr-30 | A/Hong Kong/5738/2014 | National Institute for Medical Research | Centers for Disease Control and Prevention |
| EPI551880 | HA | Hong Kong (SAR) | 2014-Aug-01 | A/Hong Kong/7278/2014 | Government Virus Unit | National Institute for Medical Research |
| EPI551878 | HA | Hong Kong (SAR) | 2014-Aug-02 | A/Hong Kong/7276/2014 | Government Virus Unit | National Institute for Medical Research |
| EPI551874 | HA | Hong Kong (SAR) | 2014-Aug-02 | A/Hong Kong/7243/2014 | Government Virus Unit | National Institute for Medical Research |
| EPI551876 | HA | Hong Kong (SAR) | 2014-Aug-04 | A/Hong Kong/7272/2014 | Government Virus Unit | National Institute for Medical Research |
| EPI551884 | HA | Hong Kong (SAR) | 2014-Aug-07 | A/Hong Kong/7313/2014 | Government Virus Unit | National Institute for Medical Research |
| EPI551882 | HA | Hong Kong (SAR) | 2014-Aug-07 | A/Hong Kong/7295/2014 | Government Virus Unit | National Institute for Medical Research |
| EPI551886 | HA | Hong Kong (SAR) | 2014-Aug-11 | A/Hong Kong/7329/2014 | Government Virus Unit | National Institute for Medical Research |
| EPI551888 | HA | Hong Kong (SAR) | 2014-Aug-14 | A/Hong Kong/7347/2014 | Government Virus Unit | National Institute for Medical Research |
| EPI551890 | HA | Hong Kong (SAR) | 2014-Aug-18 | A/Hong Kong/7364/2014 | Government Virus Unit | National Institute for Medical Research |
| EPI557298 | HA | Hong Kong (SAR) | 2014-Dec-01 | A/Hong Kong/7624/2014 | Government Virus Unit | National Institute for Medical Research |
| EPI557292 | HA | Hong Kong (SAR) | 2014-Dec-01 | A/Hong Kong/7621/2014 | Government Virus Unit | National Institute for Medical Research |
| EPI551858 | HA | Hong Kong (SAR) | 2014-Feb-16 | A/Hong Kong/4244/2014 | Government Virus Unit | National Institute for Medical Research |
| EPI653201 | HA | Hong Kong (SAR) | 2014-Feb-26 | A/Hong Kong/4801/2014 | Crick Worldwide Influenza Centre | Centers for Disease Control and Prevention |
| EPI539574 | HA | Hong Kong (SAR) | 2014-Feb-26 | A/Hong Kong/4800/2014 | Government Virus Unit | National Institute for Medical Research |
| EPI551856 | HA | Hong Kong (SAR) | 2014-Jan-12 | A/Hong Kong/1194/2014 | Government Virus Unit | National Institute for Medical Research |
| EPI718980 | HA | Hong Kong (SAR) | 2014-Jul-29 | A/Hong Kong/7127/2014 | National Institute for Medical Research | Centers for Disease Control and Prevention |
| EPI551872 | HA | Hong Kong (SAR) | 2014-Jul-31 | A/Hong Kong/7229/2014 | Government Virus Unit | National Institute for Medical Research |
| EPI551864 | HA | Hong Kong (SAR) | 2014-Jun-05 | A/Hong Kong/6033/2014 | Government Virus Unit | National Institute for Medical Research |
| EPI551862 | HA | Hong Kong (SAR) | 2014-Jun-05 | A/Hong Kong/6013/2014 | Government Virus Unit | National Institute for Medical Research |
| EPI551866 | HA | Hong Kong (SAR) | 2014-Jun-14 | A/Hong Kong/6315/2014 | Government Virus Unit | National Institute for Medical Research |
| EPI551868 | HA | Hong Kong (SAR) | 2014-Jun-19 | A/Hong Kong/6421/2014 | Government Virus Unit | National Institute for Medical Research |
| EPI540516 | HA | Hong Kong (SAR) | 2014-Mar-19 | A/Hong Kong/5383/2014 | Government Virus Unit | Centers for Disease Control and Prevention |
| EPI539578 | HA | Hong Kong (SAR) | 2014-Mar-20 | A/Hong Kong/5320/2014 | Government Virus Unit | National Institute for Medical Research |
| EPI537710 | HA | Hong Kong (SAR) | 2014-Mar-21 | A/Hong Kong/5434/2014 | Government Virus Unit | Centers for Disease Control and Prevention |
| EPI557296 | HA | Hong Kong (SAR) | 2014-Nov-29 | A/Hong Kong/7623/2014 | Government Virus Unit | National Institute for Medical Research |
| EPI565680 | HA | Hong Kong (SAR) | 2014-Nov-29 | A/Hong Kong/7615/2014 | Government Virus Unit | Centers for Disease Control and Prevention |
| EPI560450 | HA | Hong Kong (SAR) | 2014-Nov-29 | A/Hong Kong/7620/2014 | Government Virus Unit | Centers for Disease Control and Prevention |
| EPI623490 | HA | Hong Kong (SAR) | 2014-Nov-29 | A/Hong Kong/7617/2014 | Government Virus Unit | Centers for Disease Control and Prevention |
| EPI557294 | HA | Hong Kong (SAR) | 2014-Nov-30 | A/Hong Kong/7622/2014 | Government Virus Unit | National Institute for Medical Research |
| EPI551153 | HA | Hong Kong (SAR) | 2014-Oct-08 | A/Hong Kong/7563/2014 | Hong Kong Department of Health | Hong Kong Department of Health |
